# The Charter Revision Commission and the Buffalo Medical and Surgical Association

**Published:** 1889-03

**Authors:** 


The Charter Revision Commission and the Buffalo Medical and Surgical Association.—At the last meeting of the Buffalo Medical and Surgical Association, upon application of the Secretary of the Merchants' Exchange, a committee was appointed to represent the medical profession of Buffalo in the charter revision commission. Upon motion, the President was empowered to appoint the committee, which should consist of five members, one of which must be himself. The following names were selected: Drs. R. Park, W. S. Tremaine, F. W., Hinkle, John H. Pryor, and the President, Dr. P. W. Van Peyma.

It is difficult to understand how a more unrepresentative committee could possibly have been selected, had there been mature deliberation with the sole object of accomplishing such a purpose. These are all honorable gentlemen, and most excellent physicians. Four of them teach in one of the colleges, and one is an army surgeon; two of them have resided here barely more than five years; while one is a young man almost fresh from college. The President himself seems to be about the only member of the committee who can be called representative.

The complexion of the committee can only be explained on the ground that its members sought appointment thereon; and this we understand to have been the case, with one exception.

It seems to us a mistake that such men as Dr. A. H. Briggs, late Health Physician; Dr. W. C. Phelps, also a former Health Physician; Dr. Edward Clark, the present Health Physician, and Dr. E. C. W. O'Brien, who had so many years' experience in that office, were not associated with the President in the committee. Their large experience in public affairs would have been of great service to the commission. We have no doubt that the interests of the profession will be properly served by Dr. W. S. Tremaine, who has been appointed to the Executive Committee of the Commission, and who is a man of affairs in every sense of the word. The only suggestion we have to offer is that, when committees are made up from public societies for the purpose of representing the medical profession, representative men shall be chosen to serve upon them.

